# Feasibility and Safety of Pleuroscopic Cryobiopsy of the Pleura: A Prospective Study

**DOI:** 10.1155/2018/6746470

**Published:** 2018-01-22

**Authors:** Chia-Hung Chen, Wen-Chien Cheng, Biing-Ru Wu, Chih-Yu Chen, Wei-Chun Chen, Wei-Chih Liao, Chih-Yen Tu

**Affiliations:** ^1^Division of Pulmonary and Critical Care Medicine, Department of Internal Medicine, China Medical University Hospital, Taichung, Taiwan; ^2^Department of Respiratory Therapy, China Medical University, Taichung, Taiwan; ^3^Graduate Institute of Clinical Medical Science, China Medical University, Taichung, Taiwan; ^4^School of Medicine, China Medical University, Taichung, Taiwan; ^5^Department of Internal Medicine, Hyperbaric Oxygen Therapy Center, China Medical University, Taichung, Taiwan; ^6^Department of Life Science, National Chung Hsing University, Taichung, Taiwan

## Abstract

**Background:**

The aim of this study was thus to evaluate the feasibility and safety of taking biopsy specimens by cryoprobe from the parietal pleura during semirigid pleuroscope.

**Methods:**

In a single-center, observational, prospective study, patients with exudative pleural effusion (EPE) were evaluated with a semirigid pleuroscope between January 2015 and July 2017. Each patient underwent pleural biopsy using flexible forceps and flexible cryoprobe through pleuroscope following diagnostic thoracentesis and closed pleural biopsy (CPB).

**Results:**

A total of 92 patients (median age 64 years) were included in the study, most of whom were men (65.2%). Cytological cell block (CCB) and CPB made definitive diagnoses in 32/92 (34.8%) and 25/92 (27.5%), respectively; flexible forceps biopsy (FFB) and cryoprobe biopsy (CB) established definitive diagnoses in 84/92 (91.3%) and 91/92 (98.9%), respectively. The sample obtained by CB (9.4 ± 4.9 mm) was significantly larger than the other two methods: FFB (4.2 ± 2.3 mm) or CPB (1.9 ± 1.0 mm) (*P* < 0.0001). The immunohistochemical (IHC) staining was more easily performed with CB (98.9%) compared to either FFB (87.0%) or CPB (13.0%). There were no significant complications or procedure-related deaths.

**Conclusions:**

Based on these results, CB during semirigid pleuroscope has a high diagnostic yield, differentiating EPE of unknown etiology with satisfactory effectiveness and safety.

## 1. Introduction

Over fifty different pleuropulmonary or systemic disorders can cause pleural effusions. Following diagnostic thoracentesis and closed pleural biopsy (CPB), up to 25 percent of patients remain undiagnosed [1, 2]. Medical thoracoscopy increases the diagnostic yield in these cases because it offers the clinician a “window” for direct visualization and collection of samples from the parietal pleura [3]. Rigid thoracoscopy, with or without video assistance has traditionally been the procedure of choice [4]. A now widely available alternative to the conventional rigid thoracoscope is the semirigid fiber-optic video pleuroscope [5, 6], which has good sensitivity (91%) and specificity (100%) in the diagnosis of exudative pleural effusions (EPEs) and good positive and negative likelihood ratios (4.92 and 0.08, resp.) [7]. A semirigid pleuroscope can also be used therapeutically to aspirate pleural fluid or to separate pleural adhesions [8].

However, the obtained biopsy samples by semirigid pleuroscope are small and insufficient depth due to lack of mechanical power and the small size of the cup of the biopsy forceps [7, 9]. This results in concerns about diagnostic adequacy, especially in patients with significantly thickened pleura, and for this reason, new biopsy instruments are needed. Biopsy via cryoprobe, first used in 1968, has mainly been employed in the management of obstructive endobronchial tumors. Recently, cryotherapy in bronchoscopy has been used for several purposes including management of endobronchial tuberculosis [10], endobronchial tumors [11–13], for diagnosis of interstitial lung disease (ILD) [14–17], and peripheral lung lesion [18]. These studies have demonstrated that the cryoprobe is feasible and a more efficient diagnostic tool with a superior diagnostic rate and ability to obtain a larger specimen with better preserved cellular architecture than forceps biopsy.

There has been an increasing interest in the feasibility and safety of pleural cryobiopsy (CB) during semirigid pleuroscope. Several studies have shown that CB during flexi-rigid pleuroscope is feasible, safe, and effective [19–21]. However, most of these studies were conducted either with small populations or retrospectively. This prospective study thus aimed to evaluate the efficacy and safety of CB during semirigid pleuroscope in diagnosis of EPE. We also assessed the diagnostic yield of CB, quality of biopsy tissue obtained, and feasibility of immunohistochemistry (IHC) staining, in a comparison with conventional methods (CCB, CPB, and FFB).

## 2. Patients and Methods

The study was performed prospectively between January 2015 and July 2017 at the department of the Division of Pulmonary and Critical Care Medicine in the China Medical University Hospital, which is a 2,146-bed community-based university hospital in Taichung, Taiwan. The study was approved by the China Medical University Hospital Internal Review Board (CMUH103-REC1-112), and written informed consent was obtained from all patients.

### 2.1. Enrolled Patients

The patients were recruited from inpatient clinics if they met the following criteria: (1) age >18 years, (2) presence of unilateral pleural effusion of unknown origin after less invasive means of diagnosis, and (3) presence of advanced epidermal growth factor receptor (EGFR) mutation positive nonsmall cell lung cancer (NSCLC), (4) clinical resistance to an EGFR tyrosine kinase inhibitor (TKI) (gefitinib, erlotinib, or afatinib), and (5) undergoing a repeat biopsy for tumor genotyping as part of their routine clinical care. Patients with the following criteria were excluded: (1) a tendency for uncontrolled bleeding, (2) unstable cardiovascular status or severe heart failure, unstable hemodynamic status, (3) Eastern Cooperative Oncology Group performance status 4, and (4) persistent hypoxemia.

### 2.2. Instruments

**Figure 1:**
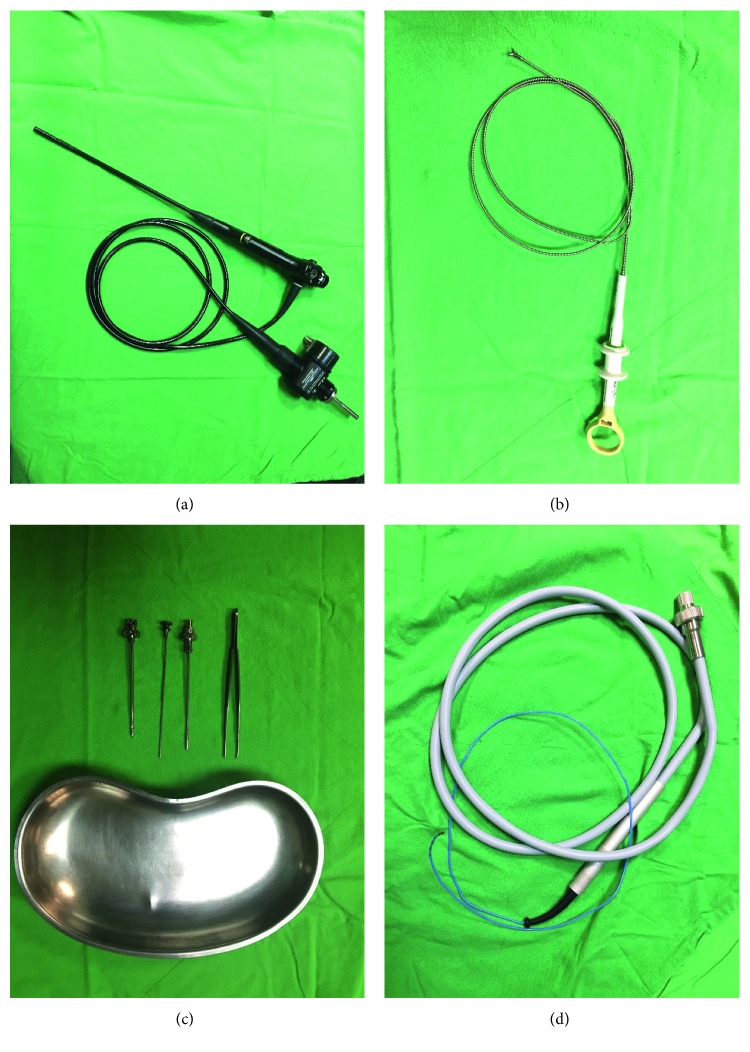
(a) Semirigid pleuroscope; (b) flexible forceps; (c) Abram's needle; (d) flexible cryoprobe.

The instrument that we used was the autoclavable Olympus LTF-240 (Olympus, Tokyo, Japan) semirigid pleuroscope with 2.8 mm inner channel diameter. FFB of the parietal pleural was taken with flexible FB-35C-1 Olympus forceps with cusps being 2.4 mm for the outer diameter and a length of 3.5 mm; the CB sample was obtained using a flexible cryoprobe with a 1.9 mm diameter and a 900 mm length (20416-037, Erbokryo CA, Erbe, Germany) and the CPB sample was sampled via Abram's needle (Figure 1).

### 2.3. Procedure

We performed diagnostic pleuroscopy in bronchoscopy suite on a spontaneous breathing patient. Bedside thoracic ultrasound was performed prior to the procedure to establish the optimal entry point. The patient was positioned in the lateral decubitus with the involved side upward. Topical anesthesia was achieved by 2% lidocaine mixed with adrenaline, applied subcutaneously and throughout the chest wall to the parietal pleura. Patients received intravenous midazolam for analgesia with sedation. Patients' blood pressure, pulse rate, and saturation were continuously monitored. Supplemental oxygen was given during the procedure. Initially, CPB with Abram's needle was performed before trocar insertion. All pleural fluid was removed after insertion of the semirigid pleuroscope, and the pleural space was thoroughly inspected. Suspected lesions in parietal pleural nodules were taken by flexible forceps biopsy and cryoprobe biopsy. Four sample specimens from CPB, forceps biopsy, and cryoprobe biopsy were taken in each patient. To avoid selection bias, we used forceps biopsy and cryoprobe pleural biopsy in the same parietal pleural nodule. A 14F chest tube was placed at the end of the procedure, and a chest radiograph was obtained afterwards. The drainage amount of pleural effusion and duration of procedures from semirigid pleuroscope insertion to final withdrawal were recorded.

### 2.4. Tissue Sampling and Complications

CPB was performed prior to the insertion of the semirigid pleuroscope. After giving local anesthesia at a suitable location at the dorsolateral thoracic wall, pleural fluid was sampled via Abram's needle, and four biopsy specimens were taken with an inward motion of the closed biopsy punch.

Conventional FFB was obtained using flexible biopsy forceps that could be accommodated within the working channel of the semirigid pleuroscope. CB was performed following the pleural biopsy using FFB. The cryoprobe tip was applied to the area of parietal pleura to be biopsied, and the cryoprobe was activated for ∼3 s by a foot-switch activation mechanism. The probe tip cooled to −89.5°C within seconds after activation. The semirigid pleuroscope and the probe, with the biopsied pleural tissue attached to it, were then withdrawn together. Each biopsy sample was released from the probe by thawing in saline, and then the sample was fixed in formalin. Four biopsy specimens were taken in all procedures.

The biopsy sites were assessed for bleeding each time before further biopsies were performed. The degree of bleeding at the biopsy site was assessed as follows: nil = slight, self-limited; mild bleeding = requiring vasoactive drug (adrenaline) injection; and moderate to severe bleeding = requiring electrocautery or argon plasma coagulation (APC) intervention.

### 2.5. Pathology and Diagnosis

The biopsy samples were processed as per standard protocol for histopathology and immunohistochemical (IHC) staining. An institutional pathologist routinely conducted the histologic analysis. For each patient, a definitive diagnosis was made on the basis of the results of the pathology.

### 2.6. Statistical Analysis

The data were analyzed using SPSS for Windows, version 17.0 (Chicago, IL, USA). Continuous variables were reported as means ± SDs and were compared using 2-tailed Student's *t*-tests. Categorical variables were reported as the numbers of patients and percentages. Differences between categorical variables were evaluated using Fisher's exact test. All statistical tests were 2 sided; a *P* value < 0.05 was considered significant.

## 3. Results

### 3.1. Characteristics of the Patients and Pleural Effusion

A total 92 patients (32 women, 60 men) with a median age of 64 years (range 22–92) were included in the study (Table 1). Eighty-three patients underwent semirigid pleuroscope for workup of an undiagnosed pleural effusion, and 9 patients with EGFR mutation NSCLC and clinical resistance to an EGFR TKI underwent repeat biopsy for tumor genotyping by semirigid pleuroscope. The samples were obtained using all techniques (CPB, FFB, and CB). Cytological cell block (CCB) was also analyzed in the diagnosis of pleural effusions. Pleural malignancy (47 lung adenocarcinoma, 1 large cell carcinoma, 2 squamous cell carcinoma, 3 lymphoma, 2 mesothelioma, 2 nasopharyngeal cancer, 2 breast cancer, 2 thyroid cancer, 2 colon cancer, 1 cholangiocarcinoma, 1 angiosarcoma, 1 gastric cancer, 1 cervix cancer, 1 sarcomatoid carcinoma, and 1 urothelial carcinoma) was diagnosed in 69 patients, and benign pleural diseases (8 tuberculosis pleuritic, 2 autoimmune pleuritic, and 13 chronic pleuritic) were diagnosed in 23 patients (Table 2). The average duration of the procedure was 34.2 min (±8.6 min), and the average drainage amount of pleural effusion was 1059 ml (±757 ml). The average duration of chest tube drainage was 2.6 days (±2.4 days), and the average length of stay after procedure was 2.4 days (±2.6 days). There were no deaths within 30 days after the procedure.

### 3.2. Diagnostic Yield and Sample Quality

**Figure 2:**
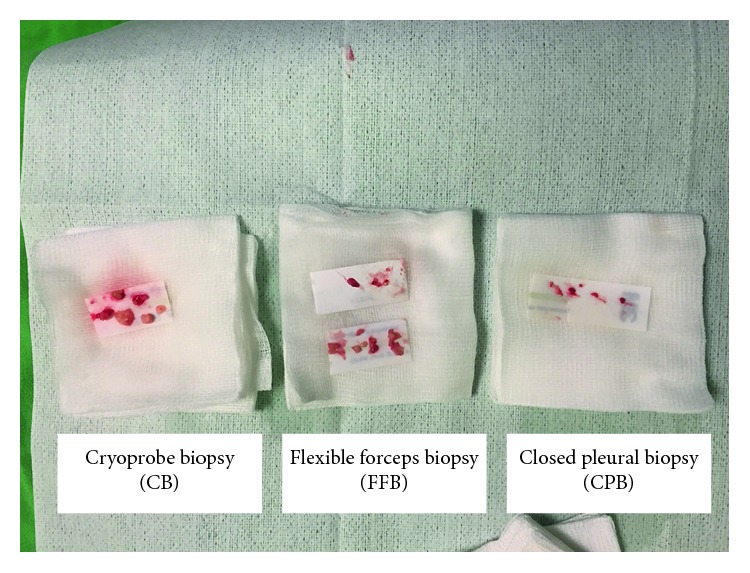
The pleural biopsy obtained by CB had larger tissue size with better tissue integrity compared to the other two methods.

**Figure 3:**
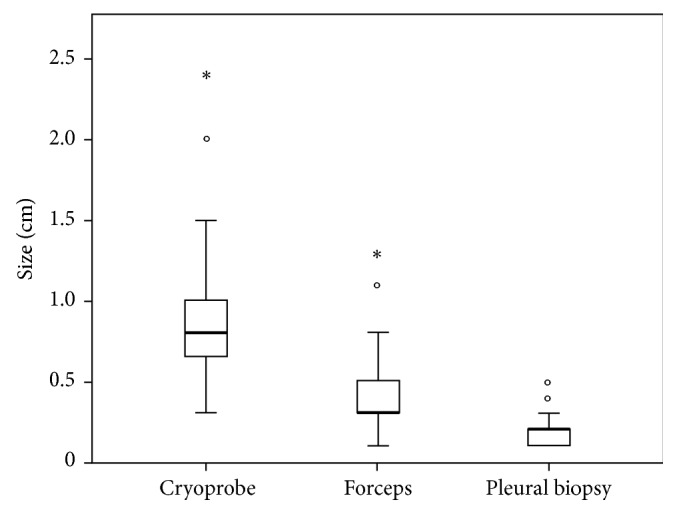
The sample obtained by CB (9.1 ± 4.5 mm) was significantly bigger than the other two methods: FFB (4.0 ± 2.1 mm) or CPB (1.9 ± 1.0 mm) (*P* < 0.0001).

The diagnostic yield was 32/92 (34.8%) in CCB. The CPB yielded a definitive histopathological diagnosis in 25/92 (27.1%) patients. FFB was able to establish a definitive diagnosis in 84/92 (91.3%) patients. A diagnosis from CB was made in 91/92 (98.9%) patients. Evaluation of the 92 patients revealed an advantage for the new technique over conventional technique, with the achieved diagnostic yields of CB being significantly higher than the other three methods of FFB, CPB, and CCB (*P*=0.017, *P* < 0.001, and *P* < 0.001 resp.). The immunohistochemical (IHC) staining was feasible in CB samples, identifying 91/92 (98.9%) patients with a definite diagnosis, which was significantly higher than that of the other three methods of FFB (80/92, 87.0%, *P*=0.001), CPB (12/92, 13.0%, *P* < 0.0001), and CCB (12/92, 13.0%, *P* < 0.0001) samples (Table 3). The sizes of specimens obtained by CB (9.4 mm ± 4.9 mm) were significantly bigger than those of the either CPB (1.9 mm ± 1.0 mm) or FFB (4.2 mm ± 2.3 mm) (*P* < 0.0001). Moreover, the CB specimens tended to be artifacts that were less crushed and had better tissue integrity (Figures 2 and 3).

### 3.3. Complications

The safety data are reported in Table 3. All the procedures were generally well tolerated by the patients. There were no significant complications reported in any patient following the procedures. Mild bleeding, requiring vasoactive drug (adrenaline) injection, was observed in both groups (6/92 patients after FFB and 8/92 patients after CB). No moderate to severe bleeding requiring electrocautery or APC intervention occurred.

## 4. Discussion

This was a prospective, single-center, controlled study evaluating the novel CB technique in comparison with conventional technique using CCB, CPB, and FB in the diagnosis of EPE during semirigid pleuroscopy. Our study showed that CB during semirigid pleuroscopy was a safe technique with a higher diagnostic yield (98.9%) for the diagnosis of EPE. In addition, IHC staining may be easily performed in samples obtained by CB (98.9%). Our explanation for this is that CB samples (9.4 mm ± 4.9 mm) are larger and have better tissue integrity than samples from the other techniques. Most importantly, there were no significant complications after CB, with the occurrence of mild bleeding after biopsy being similar in CB and FFB.

Medical thoracoscopy is a useful, effective, and safe method for investigating and managing undiagnosed EPE [22]. It can be performed using either a rigid thoracoscope or a semirigid pleuroscope [3]. Although the samples obtained by semirigid pleuroscope were smaller than those from the rigid thoracoscope, there were no differences in the quality and interpretability of the specimens assessed by the pathologist. In other words, both approaches provide comparable diagnostic yields [9, 23]. However, taking adequate samples from thickened pleura (e.g., in mesothelioma and fibrothorax) remains the most important limitation of the semirigid pleuroscope. This was not only a difficult but also time-consuming task due to the lack of mechanical power and the small size of the cup of the flexible forceps.

In addition, the histological finding of ‘nonspecific pleuritis' is common in thoracoscopic forceps biopsies, and their false-negative rate for the detection of pleural malignancy has been determined to be around 5%, with the most frequent false-negative diagnosis being mesothelioma [2, 24, 25]. The mean interval between nondiagnostic thoracoscopy and the final diagnosis was 9.8 (±4.6) months [24], and for mesothelioma, the interval was 8.7 months [26]. To overcome the limitation of small biopsies by semirigid pleuroscope, CB may be another choice. In our study, only one patient (1.1%) without definitive diagnosis which was diagnosed mesothelioma by surgery. As cancer therapy becomes more individualized, larger and better quality tissue obtained at biopsy might facilitate mutational analysis and genetic profiling [27]. Therefore, 9 patients with EGFR mutation lung adenocarcinoma and having clinical resistance to an EGFR TKI underwent repeat biopsy for tumor genotyping by cryoprobe biopsy during semirigid pleuroscopy. CB successfully obtained pleural tissue suitable for histopathological analysis in all 9 patients.

Cryotherapy in bronchoscopy has been used for diagnosis and management of endobronchial lesion [11–14, 28, 29]. Endobronchial CB is safe and increases the diagnostic yield in comparison with conventional FB [11, 30, 31]. The overall superiority of CB is most likely due to the higher quality of the samples from their significantly larger biopsies regarding size and artifact-free tissue sections, which has been shown in previous studies [14, 28, 31, 32]. The cryotherapy probe also facilitates sampling of target lesions positioned tangentially to the instrument, which are difficult than samples from flexible forceps, especially in narrow airway. The potential advantages of this method are the shortening of the duration of the procedure and avoidance of repeated biopsy-related complications [30].

A number of recent studies have assessed the feasibility and safety of pleural CB during semirigid pleuroscopy. These results are summarized in Table 4. Rozman et al. demonstrated that the samples were of good quality (81% were easily interpretable), with the level of artifacts below 25%. The specimens were adequate for histological diagnosis, IHC staining, and DNA isolation, and a definitive diagnosis was achieved in 20/22 (90%) of the subjects undergoing CB and FFB [20]. Thomas et al. reported that CB (10 mm, 7–15.8) was larger than FFB (4 mm, 3–8) and had better preserved cellular architecture and tissue integrity. Crushed artifacts were less common with CB (2/22) compared with FFB (21/22), and both FFB and CB made definitive diagnoses in 15/15 (100%) and 14/14 (100%) [19]. Maturu et al. also showed that CB was significantly larger (*P*=0.001) when compared with FFB (9.17 ± 1.84 versus 3.75 ± 0.96 mm), and that crushed artifacts were more frequent with FFB as compared with CB (3/4 versus 0/6). Similarly, the depth of tissue was greater with the cryoprobe. It revealed a higher diagnostic yield for CB (6/6) compared to FFB (3/4) [21]. The present study demonstrated that CB was significantly larger (*P* < 0.0001) when compared with FFB (9.1 ± 4.5 mm versus 4.0 ± 2.1 mm). IHC stains were more easily performed with CB as compared with FFB (91/92 versus 80/91), though not reaching statistical significance. CB has proven to have excellent tissue quality for IHC [28], and we found that the diagnostic yield was higher (but not statistically significant) with CB (50/51) than with FFB (47/51). In the case of the accuracy of diagnosis, it is well known that quality of the specimen is important. Although CB reduces the histological finding of “nonspecific pleuritic,” which presents a management dilemma for clinicians, the diagnostic yield of CB was comparable to FFB based on the abovementioned studies. These findings may be explained by the fact that these studies were small and thickened pleura pathologies were lacking in two of the studies.

In spite of the above-favorable evidence, the cryotherapy probe has not been routinely used as a biopsy instrument mainly because of concerns that the immediate pulling of a frozen probe could lead to significant complications, such as bleeding. Rozman et al. reported the assessed degree of bleeding was slight and self-limited, as expected after biopsy and no further interventions were needed [20]. Thomas et al. indicated that mild bleeding was observed in their FFB (4/22) group and CB (5/22) group [19]. Our study also indicated that the incidence of mild bleeding was similar between the CB group (8/92) and FB group (6/92). We did not observe any other complications. These findings also accord with previous studies [19, 20] that found CB during flexi-rigid pleuroscopy was safe.

In spite of our careful study design, we acknowledge a number of limitations in our study. First, only 92 patients were enrolled in our study in a single hospital. Second, the CB was taken after conventional FFB. Bleeding may not be fairly evaluated when samples from the same lesion were taken by CB and FFB. Third, for patients with thickened pleural pathologies where FFB biopsy alone might lead to a false-negative result, no additional diagnostic benefits were found for CB over conventional FFB; one explanation for this is that subjects with mesothelioma or fibrotic pleural pathologies were lacking in our study.

## 5. Conclusions

The results of the present study may be summarized as CB during semirigid pleuroscope is a safe and feasible technique and it has a diagnostic yield for the diagnosis of undiagnosed EPE that is comparable to conventional pleural biopsy using FFB. CB was also easier to interpret by the pathologist because of the larger tissue samples with better preserved cellular architecture. Larger randomized trials and multicenter studies are required for further evaluation of the routine use of CB during semirigid pleuroscopy.

## Figures and Tables

**Table 1:** Characteristics of the patients.

Characteristics	Medical pleuroscopy patients (*n* = 92)
Age	64.8 ± 13.9
Sex (*n*, %)	
Male	60 (65.2)
Female	32 (34.8)
Underlying disease (*n*, %)	
None	35 (38.0)
Malignancy	26 (28.3)
DM	11 (12.0)
ESRD	5 (5.4)
CHF	5 (5.4)
Reason for medical pleuroscopy (*n*, %)	
Unknown pleural effusion	83 (90.2)
Rebiopsy	9 (9.8)

**Table 2:** Characteristics of the pleural effusion.

Characteristics	Medical pleuroscopy patients (*n* = 92)
Etiology for pleural effusion	
Malignancy	69 (75)
Lung adenocarcinoma	47 (51.1)
Lung large cell carcinoma	1 (1)
Lung squamous cell carcinoma	2 (2.2)
Lymphoma	3 (3.3)
Mesothelioma	2 (2.2)
Nasopharyngeal cancer	2 (2.2)
Breast cancer	2 (2.2)
Thyroid cancer	2 (2.2)
Colon cancer	2 (2.2)
Sarcomatoid carcinoma	1 (1)
Cholangiocarcinoma	1 (1)
Cervix cancer	1 (1)
Urothelial carcinoma	1 (1)
Gastric cancer	1 (1)
Angiosarcoma	1 (1)
Benign	23 (25)
TB	8 (8.7)
Autoimmune related	2 (2.2)
Chronic pleuritic	13 (14.1)
Drainage amount (ml)	1059 ± 757
Duration of procedure (minutes)	34.2 ± 8.6
Duration of subsequent chest drainage (days)	2.6 ± 2.4
Length of stay after procedure (days)	2.4 ± 2.6
30 days mortality	0

**Table 3:** The diagnostic yield, size, and quality of samples and complications.

Semirigid pleuroscope patients (*n* = 51)	Close pleural biopsy	Flexible forceps biopsy	Cryoprobe biopsy	Cytology cell block
Diagnostic yield	25 (27.1)	84 (91.3)	91 (98.9)	32 (34.8)
For IHC stain	12 (13.0)	80 (87.0)	91 (98.9)	12 (13.0)
Size, mm	1.9 ± 1.0	4.2 ± 2.3	9.4 ± 4.9	—
Complication				
Bleeding				
Nil	92	86	84	0
Mild	0	6	8	0
Moderate to severe	0	0	0	0

Nil bleeding: slight, self-limited; mild bleeding: needing bosmin injection; moderate to severe: needing coagulation or APC. *P* value of diagnostic yield: close pleural biopsy versus flexible forceps biopsy: <0.0001; close pleural biopsy versus cryoprobe biopsy: <0.0001; close pleural biopsy versus cytology cell block: 0.929; flexible forceps biopsy versus cryoprobe biopsy: 0.017. *P* value of IHC stain: close pleural biopsy versus flexible forceps biopsy: <0.0001; close pleural biopsy versus cryoprobe biopsy: <0.0001; close pleural biopsy versus cytology cell block: 0.998; flexible forceps biopsy versus cryoprobe biopsy: 0.001. *P* value of size: close pleural biopsy versus flexible forceps biopsy: <0.0001; close pleural biopsy versus cryoprobe biopsy: <0.0001; flexible forceps biopsy versus cryoprobe biopsy: <0.0001.

**Table 4:** Comparison with similar studies.

Medical pleuroscopy patients (*n *= 51)	Rozman et al. [19]	Thomas et al. [18]	Maturu et al. [20]	Our study
Number of subjects	15	22	6	92
Age	61 (33–83)	72 (47–89)	50 (29–61)	64 (22–92)
Sex (male : female)	12 : 3	21 : 1	4 : 2	2 : 1
Quality of sample				
Size (mm) (CB versus FB)	NA	10 versus 4	9.2 versus 3.7	9.4 versus 4.2
Artifacts on histology				
CB	6/14	2/22	0/6	NA
FB	NA	21/22	3/4	NA
Diagnostic yield				
CB	14/14	20/22	6/6	91/92
FB	15/15	20/22	3/4	84/92
Final diagnosis of mesothelioma	9	11	0	0
Bleeding (CB versus FB)				
Nil	0	17 versus 18	NA	84 versus 86
Mild	14 versus 15	5 versus 4	NA	8 versus 6
Moderate to severe	0	0	0	0

NA: not available.

## Abbreviations

EPE: Exudative pleural effusion
CB: Cryoprobe biopsy
CPB: Closed pleural biopsy
CCB: Cytological cell block
FFB: Flexible forceps biopsy
IHC: Immunohistochemical
APC: Argon plasma coagulation
ILD: Interstitial lung disease
NSCLC: Nonsmall cell lung cancer
EGFR: Epidermal growth factor receptor
TKI: Tyrosine kinase inhibitor.
